# A Dual-Purpose Biomedical Measurement System for the Evaluation of Real-Time Correlations Between Blood Pressure and Breathing Parameters

**DOI:** 10.3390/s26020452

**Published:** 2026-01-09

**Authors:** José Dias Pereira

**Affiliations:** 1 Instituto Politécnico de Setúbal, Escola Superior de Tecnologia de Setúbal Campus do IPS, Estefanilha, 2910-761 Setúbal, Portugal; dias.pereira@estsetubal.ips.pt; 2 Instituto de Telecomunicações, Av. Rovisco Pais, 1049-001 Lisbon, Portugal

**Keywords:** blood pressure and breathing parameters, measurement system, calibration, accuracy, bio-signal processing, cross-correlation

## Abstract

This paper proposes a low-cost measurement system that can be used to perform simultaneous blood pressure (BP) and breathing (BR) measurements. Regarding BP measurements, the main parameters that are accessed include systolic blood pressure (SBP), diastolic blood pressure (DBP), mean arterial pressure blood pressure (MAP), and heartbeat rate (HR). Concerning BR measurements, the main parameters that are accessed include the inspiration period and amplitude (IPA), the expiration period and amplitude (EPA), and the breathing rate (BR), as well as the statistical and standard deviation of all these parameters. The dual measurement capability of the proposed measurement system is very important since blood pressure and breathing parameters are not statistically independent and it is possible to obtain additional and valuable clinical information from the information provided by both biomedical variables when measured simultaneously. The analysis of the correlation between these variables is particularly important after performing intensive physical exercises, since it enables cardiac rehabilitation assessment, pre-surgical risk evaluation, detection of silent ischemia, and monitoring of chronic diseases recovery, among others. Regarding the performance evaluation of the proposed biomedical device, a prototype of the measurement system was developed, tested, and calibrated. Several experimental tests were carried out to evaluate the performance of the proposed measurement system and to obtain the correlation coefficients between different blood pressure and breathing parameters. The tests were based on a statistically significant number of measurements that were performed with a population that integrated twenty students in two groups with different habits of physical exercise practice but subjected to a set of common physical exercises, with graduated intensity levels.

## 1. Introduction

Besides the well-known importance of blood pressure (BP) and breathing (BR) measurements, it is important to note that if both measurements are performed simultaneously, it is possible to access additional and valuable clinical information. An example of the importance of the simultaneous measurements of both variables occurs when BP and BR parameters are analyzed in real time after physical exercise practice. Abnormal recovery of BR and BP parameters after the practice of physical exercises, namely, delayed normalization of breathing rate or delayed normalization of systolic blood pressure values, can indicate different types of diseases such as chronic obstructive pulmonary diseases, asthma, interstitial lung disease, exercise-induced hypertension, and autonomic dysfunction, among others. Moreover, an excessive rise in systolic BP or a delayed return to baseline values may indicate the existence of potential heart failure causes, coronary artery diseases, or poor left ventricular function. On the other hand, a sudden drop in BP values after stopping physical exercises can be caused by a nervous system dysfunction, such as Parkinson’s or diabetic neuropathy [1]. Thus, the usage of combined measurements of BP and BR parameters gives a more complete picture of cardiopulmonary fitness and helps the identification of potential health risks. The trends of BP and BR parameters after the practice of physical exercises can also be used to control and individualize physical exercise intensity levels based on breathing and blood pressure variations. It is also important to note that regarding the state of the art, there are other scientific works that have focused on different measurement solutions that can be used to measure the heartbeat rate and the breathing rate. Some of these measurement solutions consider the usage of a sensitive microbend multimode fiber-optic sensor [2,3,4]. The main limitations of these measurement solutions are related to the difficulty of accurately measuring heart rate and breathing rate parameters using a simple frequency-domain analysis. Other limitations are related to sensor positioning stability, which affects measurement system repeatability and accuracy. It is also important to underline that these measurement solutions only access heart rate and breathing rate and no other important parameters that are related to BP and BR are accessed. Other authors have presented a measurement solution [5] that uses an extremely sensitive optical fiber microbend sensor for heart rate, breathing rate (RR), and ballistocardiography (BCG) monitoring. Besides the limitations already mentioned, it is important to note that these measurement solutions require an individual calibration for each user because the mapping between the optical strain and physiological signal is inconsistent due to the variations in body morphology, tissue stiffness, and breathing style of different subjects. Other authors have proposed an alternative device with a microelectromechanical system (MEMS)-based pressure sensor that can simultaneously measure the blood pulse wave and the breathing rate using only one sensing element [6]. Regarding this measurement solution, the authors claim that the pressure sensor they used can measure a differential pressure as small as 0.01 Pa. However, the calibration solution they presented only includes the sensing device and not the complete measurement chain that also includes a Wheatstone bridge circuit and an amplifier, which surely limits system accuracy significantly. Table 1 details a summary of accuracy, cost, comfort, and complexity integration for fiber-optic-, MEMS-, and pneumatic-based solutions.

Thus, to continue research activities, this paper presents a dual-purpose biomedical device with auto-testing and calibration capabilities. Calibration of the measurement chain, and not only of the sensing device, can be performed by using BP and BR calibrators that can be connected to the measurement system without requiring a different system setup than the one used for measurement purposes. This paper also includes different simulation and experimental results of studies that were conducted with two groups of students that practiced a common set of physical exercises. One of the main advantages of the pneumatic sensing-based solution proposed in this paper is related to its accuracy and easy calibration. The standard oscillometric method used for BP measurements is frequently used for clinical purposes and there are a lot of calibrators and simulators commercially available. The accuracy and sensitivity of pressure measurements using respiration belts is also very acceptable for the required pressure measuring range. Moreover, the proposed pneumatic measurement solution shares several pneumatic devices, namely the EPPR and valves, that make measurement system implementation cheaper, and the feedback nature of the pneumatical channels minimizes air leakage risks, which potentially affect measurement accuracy, and provides easy implementation of self-testing and auto-calibration capabilities. Moreover, the significant number of experimental tests that were performed validate the performance of the measurement system and its ability to access several BP and BR parameters and evaluate, in real time, the correlation between any set of measured parameters. Concerning text organization, Section one of the paper includes the introduction, Section two includes the presentation of the main parameters and methods that are used to obtain BP and BR parameters, Section three presents the hardware and software descriptions of the measurement system, Section four presents some simulation and experimental results, and the last section, Section five, presents the main conclusions of the paper.

## 2. BP and BR Parameters and Cross-Correlations

This section presents the main BP and BR parameters that can be accessed by the proposed measurement system and identifies the main correlations that exist between BP and BR parameters.

### 2.1. BP and BR Parameters

**Figure 1:**
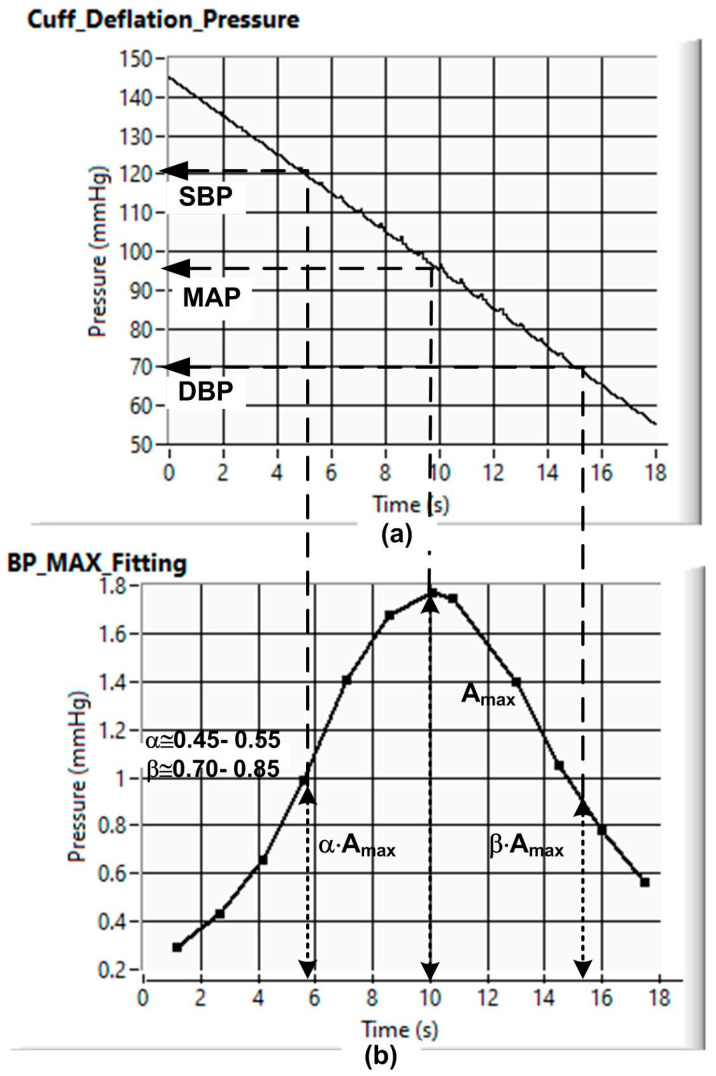
Data used to evaluate BP parameters by using the fixed-ratio oscillometric method: (**a**) pressure variation during the cuff deflation phase (MAP—mean arterial pressure; SBP—systolic blood pressure; DBP—diastolic blood pressure); (**b**) pressure peaks’ envelope caused by BP variations (Amax—maximum value of the peaks’ envelope curve; α—systolic coefficient; β—diastolic coefficient).

Regarding the main BP parameters that are used to evaluate people’s health condition, the systolic blood pressure (SBP) and the diastolic blood pressure (DBP) are the most important ones. The pressure unit that is commonly used to quantify the values of these parameters is mmHg, which corresponds to the relative pressure value generated by a column of mercury with a given number of millimeters. In a simple way, SBP represents the pressure inside the arteries when the heart contracts and indicates how much force per surface unit the blood exerts on the artery walls during each heartbeat. On the other hand, the DBP represents the pressure in the arteries when the heart is resting between beats and reflects the resistance of the blood vessels. Related to these two parameters is another frequently used BP parameter known as the mean arterial pressure (MAP), which is a good indicator of the blood perfusion capability to vital organs. The formula that establishes the relationship between MAP and the previous two BP parameters is given by

(1) $$\mathrm{MAP}=\mathrm{DBP}+\left[\left(\mathrm{PP}\right)/3\right]$$

where PP, known as pulse pressure, represents the difference between the systolic and diastolic pressures. Besides the BP parameters previously referred to, all of which relate to BP intensity values, there is another crucial heart parameter, related to the rhythmic time variations in BP values. This parameter is the heartbeat rate (HBR), which is typically expressed by the number of heartbeats per minute. It is important to note that the human body automatically controls the HBR, which increases when we make physical efforts, for example, when we are running or when we are excited or scared; conversely, the HBR drops when we are resting, calm, or comfortable. The BP parameters, namely, the MAP, the SBP, the DBP, and the HBR, were evaluated using the fixed-ratio oscillometric method [7,8,9,10]. As represented in the upper graphic of Figure 1, during the cuff deflation phase, there are very small pressure oscillations caused by BP variation. By extracting this pressure oscillation from the main pressure signal, it is possible to obtain the pressure peak amplitudes, which, in turn, are used to obtain, using curve fitting, the peak’s envelope function represented in the lower graphic of Figure 1. Then, using amplitude ratio coefficients, α and β, whose values are typically equal to 0.5 and 0.8, it is possible to estimate the SBP and DBP, respectively, from the cuff deflation pressure values associated with α·Amax and β·Amax, Amax being the maximum amplitude value of the peaks’ envelope curve associated with the MAP. Moreover, from the time indexes that are associated with the different oscillation peaks, it is possible to obtain the different parameters associated with the HBR.

**Figure 2:**
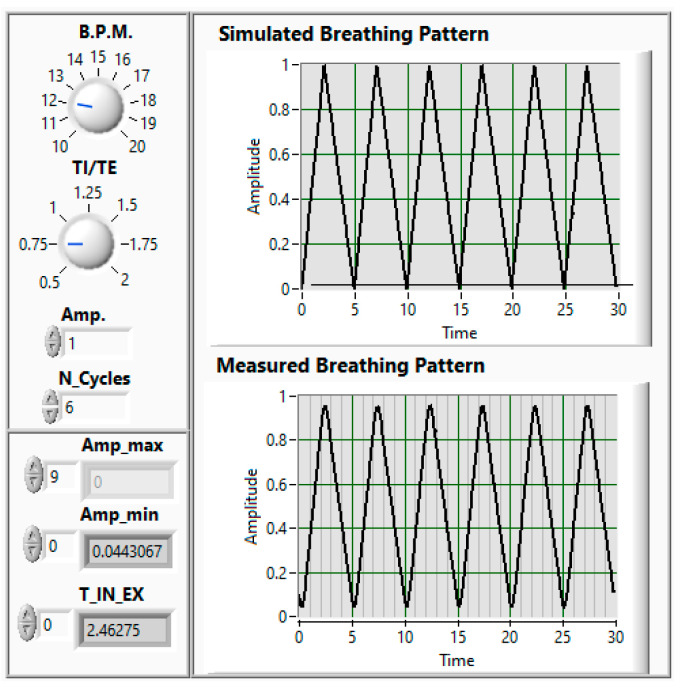
Front panel of the BR simulator (BR_R = 12 b.p.m.; TI/TE = 0.75; IP = 1; and N_Cycles = 6).

Regarding the main BR parameters that are used to evaluate people’s health condition, they include the breathing rate (BR_R), which corresponds to the number of breaths per minute; the tidal volume (TV), which corresponds to the amount of air inhaled or exhaled in a normal breath; the minute ventilation (VE), which corresponds to the total volume of air entering (or leaving) the lungs per minute, which can be calculated by the product of the BR and TV values; and the oxygen saturation (SpO2), which corresponds to the percentage of hemoglobin binding sites occupied by oxygen. Obviously, regarding clinical purposes, the number of BP and BR parameters that can be considered can be much higher; however, for monitoring purposes, that is, the aim of the proposed measurement system, the number of BP and BR parameters that are required is not so high. Thus, the BP parameters that are considered in the present paper are the systolic, diastolic, and mean arterial pressures and the heartbeat rate, and the BR parameters that are considered are the breathing rate (BR_R), the inhalation peak (IN_P), the inhalation duration (IN_D), the exhalation peak (EX_P), and the exhalation duration (EX_D). Auxiliary parameters, such as the inhalation–exhalation durations and amplitude ratios, IETR and IEAR, respectively, are also considered for data analysis. The BR parameters, namely, the BR_R, the IN_P, the IN_D, the EX_P, and the EX_D, as well as secondary ratio parameters IETR and IEAR, were evaluated by using MATLAB’s (2020a) peak-detection function. This function is important because it turns noisy waveforms into physically meaningful, measurable events, enabling accurate diagnosis, monitoring, and decision-making, which is essential in biomedical data processing. Figure 2, as an example, represents the front panel of the breathing simulator that is used to calibrate the BR measuring chain. The electrical signal, which simulates the breathing pattern, is applied to the EPPR and its pressure output is applied to the BR measuring chain, which delivers the measured breathing pattern. The main input parameters of this simulator include the BR_R, the TI/TE ratio, the amplitude of the inhalation peaks, and the number of BR cycles to test.

### 2.2. BP and BR Cross-Correlations

Many biomedical parameters are interdependent and sometimes it is difficult to properly evaluate people’s health conditions using only individual indicators. For example, blood pressure (BP) and respiratory parameters are tightly linked because the cardiovascular and respiratory systems work together to maintain oxygen delivery to and carbon dioxide removal from our body. Regarding the mechanical interaction between BR and BP parameters, during inspiration the intrathoracic pressure falls and the venous return increases, which could lead to systolic BP fluctuations. On the other hand, during expiration the intrathoracic pressure rises and the systolic BP may fall slightly. Additionally, there is an interaction associated with the natural variation in HBR that occurs with breathing, where the HBR speeds up during inhalation and slows down during exhalation, this interaction being known as respiratory sinus arrhythmia. Another BP parameter that interacts with BR parameters is related to the level of oxygen tissue perfusion, which directly affects the delivery of oxygen and nutrients to human body cells. If BP, particularly MAP, falls too low, gas exchange suffers due to inadequate pulmonary perfusion, which increases almost all the BR parameters’ values. Breathing exerts a direct modulatory effect on blood pressure (BP) parameters. Variations in intrathoracic pressure during inspiration and expiration influence venous return, while gas exchange exerts a chemical effect on BP through activation of chemoreceptors and an autonomic nervous system response. In turn, adequate blood pressure is necessary to maintain sufficient pulmonary perfusion, a prerequisite for effective gas exchange in the lungs [11]. The relevance of simultaneous measurement of BP and breathing rate (BR) is well-illustrated in cardiopulmonary exercise testing (CPET). CPET evaluates clinical indicators that are crucial for identifying unexplained physical exercise intolerance, diagnosing and monitoring cardiovascular and respiratory disorders, and assessing physical fitness levels for rehabilitation. Within the context of BP analysis, the heart/breathing rate ratio (HBRR)—calculated as heart rate divided by breathing rate—represents a valuable physiological marker. Elevated HBRR values have been associated with several pathological states, including predisposition to heart failure.

## 3. Measurement System

The measurement system includes two main parts, the hardware part and the software part. Regarding the hardware part, there are two main sensing and conditioning units. One of them implements the pressure measuring chain of the breathing signal and the other unit implements the pressure measuring chain of the blood pressure signal. The sensing units that are used to translate the physical pressure signals to the electrical domain are pneumatic pressure sensors in both cases. There are several advantages associated with the usage of pneumatic sensing in biomedical applications. The main advantages of using pneumatic sensing can be described as follows: its non-invasive nature, since there is no need for skin electrodes or invasive procedures to perform signal acquisition; its easy application and simple setup; and its intrinsic galvanic isolation between the signal conditioning circuits and the human body. Regarding disadvantages, these include its indirect measuring nature, its sensitivity to movement artifacts, and its reduced sensitivity in shallow breathing conditions that cause small breathing excursions. It is also important to note that regarding the typical bandwidth limitations of pneumatic sensing and pneumatic signal transmission [12], this limitation does not affect measurement system accuracy, since for a maximum limit of 25 breaths per minute and for a maximum heartbeat rate limit of 250 beats per minute, the associated frequencies are 0.4 Hz and 4 Hz, respectively, these values being much lower than the bandwidth of the two measurement channels of the measurement system.

### 3.1. Hardware

**Figure 3:**
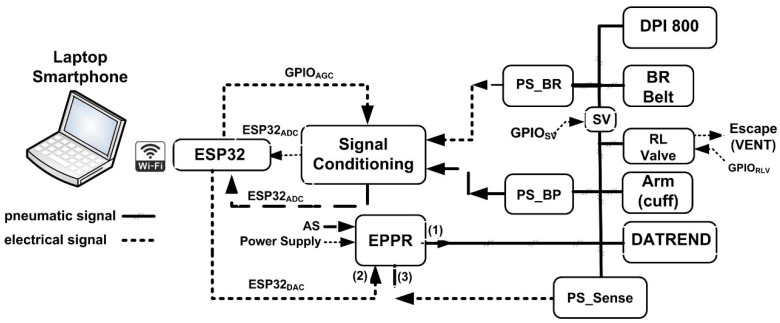
Hardware block diagram of the measurement system (AGC—automatic gain control; PS_BP—pressure sensor for BP measurements; PP_BR—pressure sensor for BR measurements; DPI 800—pressure calibrator; DATREND—NIBP calibrator; EPPR—electro-pneumatic pressure regulator; SV—solenoid valve; RLV—release valve; AS—air supply; GPIO—general purpose input/output).

Figure 3 represents the hardware block diagram of the measurement system. The hardware block diagram mainly includes two measuring chains: one is associated with BP measurement, and the other is associated with BR measurement. The BP signal is acquired by a common arm cuff [13], and a pressure sensor (PS_BP) [14] is used to measure the cuff pressure variation. By using the oscillometric method, it is possible to obtain different BP parameters, such as the systolic and diastolic BP and the heartbeat rate, among others. On the other hand, the BR signal is acquired by a breathing rate belt [15] that senses pressure variations caused by the expansion and contraction of a person’s chest during breathing. The pressure sensor (PS_BR) [16] has a differential pressure range of ±10 kpa and the voltage output signal of this pressure sensor is processed to obtain the different breathing parameters. Regarding BP measurement, the main characteristics of the pneumatic pressure sensor include a measuring range between 0 and 50 kPa, a typical full-scale (FS) output voltage equal to 2.8 V when a 3 V power supply is used, an FS span equal to 2.7 V, an accuracy better than ±2.5% of the voltage FS span, a typical sensitivity of 54 mV/kPa for the same power supply voltage of 3 V, and a response time lower than 1 ms. This set of specifications complies completely with the application requirements for BP measurement. Regarding BR measurement, the main characteristics of the pressure sensor include a measuring range between 20 and 200 kPa, a typical FS output equal to 40 mV when a 10 V power supply is used, linearity and hysteresis errors lower than 0.4% and 0.1% of the FS span, respectively, a typical sensitivity of 0.2 mV/kPa, and a response time lower than 1 ms. This set of specifications also complies completely with the application requirements for BR measurement. Since, in this case, there is a low amplitude variation in the sensor’s output voltage, the signal conditioning unit must include amplification capabilities, which is not required for the BP measuring chain. As represented in Figure 3, there is a control line (GPIO_ADC_) of the Arduino device [17,18] that enables the selection of one of two amplification gains that are equal to 100 or 200, according to the conditioning circuit output voltage level. With this setup it is possible to optimize the BR measurement sensitivity and resolution. The microcontroller ESP32 includes WiFi and Bluetooth connection capabilities that can be used to connect the measurement system to a wirelessly connected laptop, smartphone, or cloud. One crucial device that is included in the pneumatic part of the measurement system is the electro-pneumatic pressure regulator (EPPR) [19], which generates a pneumatic signal (EPPR_1) whose pressure amplitude is controlled by an electrical voltage signal (EPPR_2). The main characteristics of this device include analog or digital control, analog feedback of the output pressure signal, accuracy better than ±0.25% of FS, and repeatability and stability, both better than ±0.2% of FS. With this miniature digital pressure controller, it is possible to generate a pneumatic signal using an arbitrary waveform voltage generated by the DAC output (ESP32_DAC_) of the ESP32 microcontroller. The EPPR also includes an external pressure input port (EPPR_3) that can be used to sense the pressure applied to the pneumatic loads, in our case the BP cuff and BR belt. In this way, by using a remote pressure sensor (PS_Sense), both pneumatic measuring channels are calibrated, and small but inevitable pneumatic gas leakages between tubing and terminal connectors can be compensated for. Thus, the pneumatic calibration loop, implemented with the remote pressure sensor, assures that the calibration is not affected by the characteristics of the pneumatic silicone tubing and other pneumatic load effects [12], such as the ones that are associated with other pneumatically connected devices, including the pressure and BP calibrators. This is an important feature which concerns the accuracy of pneumatic measurement systems, since the calibration is not affected by specific characteristics of the pneumatic measurement channel, such as the ones that are associated with the length and diameters of the tubes that are used to connect the BP cuff and BR belt to the associated pressure sensors. The hardware block diagram also includes a pressure indicator [20], with an accuracy better than 0.1% of the measuring span; a non-invasive blood pressure simulator [21]; a release valve controlled by a general purpose input/output ESP32 programmable pin (GPIO_RLV_), which is mainly used to depressurize the cuff and the respiratory pneumatic belt after measurement phases; and a miniature pneumatic solenoid valve (SV) [22] that is used to decouple the pneumatic BP and BR measuring channels and controlled by another general purpose input/output ESP32 programmable pin (GPIOSV). The pneumatic decoupling of the BR and BP pneumatic channels is essential to performing both measurements simultaneously without mutual interference, since after inflating the BR belt, the solenoid valve opens and the pressure sensors, PS_BR and PS_BP, are only sensitive to pressure variations caused by breathing and blood pressure, respectively. It is also important to note that auto-testing of the measurement system can be easily performed by closing the solenoid valve and applying the same pneumatic signal, delivered by the EPPR, to both BR and BP pressure sensors.

### 3.2. Software

**Figure 4:**
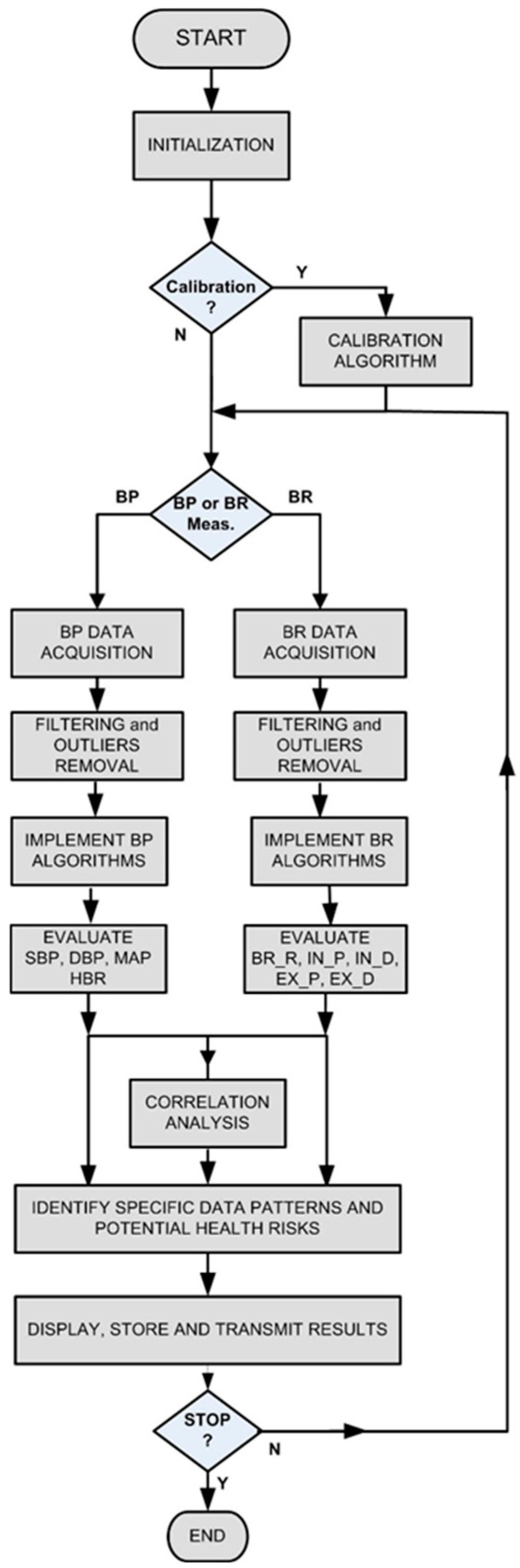
Program flowchart (BP—blood pressure; BR—breathing; SBP—systolic blood pressure; DBP—diastolic blood pressure; MAP—mean arterial blood pressure; HBR—heartbeat rate; BR_R—breathing rate; IN_P—inhalation period; EX_P—exhalation period; IN_D—inhalation depth; EX_D—exhalation depth).

Regarding software development strategy, processing routines with higher processing load, running in the laptop, were developed using a graphical programming language [23], while the routines with lower processing load, running in the microcontroller, were developed in MicroPython (V1.26.1) [24,25]. Figure 4 represents the flowchart of the software that was developed to process measurement system (MS) data. The developed software includes data processing routines to extract BP parameters and routines to extract BR parameters. The BP estimation algorithm which was implemented is based on the oscillometric method [7,8,26,27]. From the pressure oscillation peaks, the mean arterial pressure (MAP), the SBP, and the DBP were calculated by using specific algorithms [28,29]. HBR was obtained using the time durations between the oscillometric pulse peaks. Regarding the estimation of the BR parameters, a common peak-detection function [28] was also used to obtain BR_R, IN-P, IN-D, and the correspondent exhalation parameters. It is important to note that after BP and BR data acquisition, suitable filtering functions and outlier removal algorithms were used to improve the signal-to-noise ratio associated with the measurement data. The developed software also includes routines to analyze the measurement data, namely, to detect abnormal blood pressure and breathing patterns, to evaluate the correlation between BP and BR parameters, and to display, store, and transmit those parameters to a centralized data center. From the correlation analysis, between the BP and BR parameters, it is possible to identify potential health risks of a given subject under analysis. It is also important to note that particular care in data signal processing must be taken with the usage of filters and other processing operators to minimize errors caused by distortions in signal amplitudes or phases. Regarding this issue, it is important to underline that the filtering and smoothing of the oscillometric waveform, associated with BP parameter extraction, was performed using the specific smoothing filters [29].

**Figure 5:**
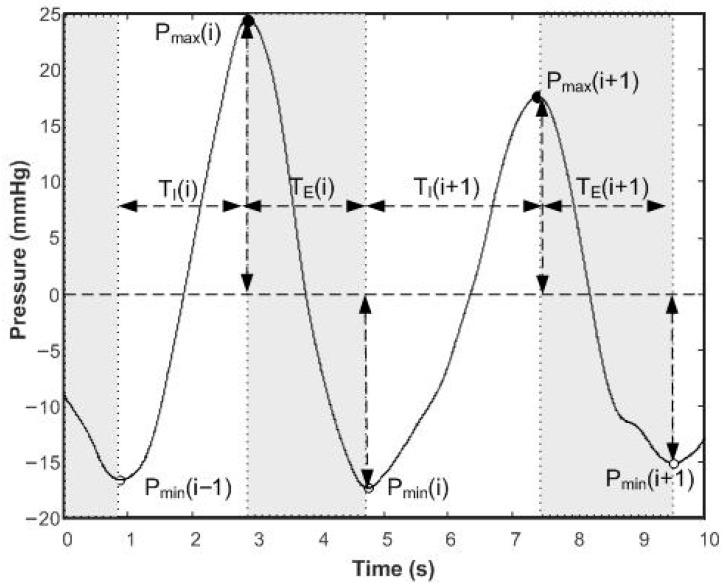
Pressure signal variations in a generic BR signal (P_min_—minimum pressure value at the end of an expiration phase; P_max_—maximum pressure value at the end of an inspiration phase; T_E_—duration of the exhalation; and T_I_—duration of the inhalation).

To evaluate BP measurement accuracy, a commercial NIBP simulator [21] was used. The main specification of this simulator includes a set of pre-defined oscillometric BP signals with different values of SBP, DBP, and HBR. The HBR values can vary in the range between 20 and 240 b.p.m. with an accuracy of ±0.25 b.p.m., and the BP values that are simulated can vary in the range between 10.0 and 400.0 mmHg, with an accuracy of ±0.5 mmHg and a resolution of 0.1 mmHg. To evaluate the BR measurement accuracy, a BR pressure signal generator was developed. To simulate the BR signal, the input control of the EPPR was connected to the DAC output (ESP32_DAC_) of the ESP32 microcontroller, making it possible to simulate different amplitude and timing intervals for the simulated BR signal. Figure 5 represents a typical simulated BR signal that is characterized by the following set of parameters: P_min_ and P_max_, which are the minimum and the maximum pressure values associated with the end of the expiration and inspiration phases, respectively, and TE and TI, which are the duration of the expiration and inspiration periods, respectively. By defining the values of the previous coefficients for each BR cycle and the inspiration plus expiration phases, it is possible to control different parameters of the BR signal, namely, the breathing rate (BR_R), the BR depth (BRD), the BR symmetry (BRS), and the breathing regularity (BRRg) [30].

The breathing parameters were defined by the following relationships:

(2) $$\begin{array}{c} \mathrm{BR}\text{\_}R\;=\;\frac{60}{T_{I}+T_{E}}\\ \begin{array}{c} \;\mathrm{BRS}\;=\;100\cdot \frac{T_{I}}{T_{I}+T_{E}}\%\\ \mathrm{BRD}\;=\;\frac{P_{\max}\;-\;P_{\min}}{P_{\mathrm{av}}}\\ \mathrm{BRRg}\;=\;\sqrt[{2}]{{\left(std(T_{I}\right))}^{2}+{\left(std(T_{E}\right))}^{2}} \end{array} \end{array}$$

where P_av_ represents the average value of the pressure signal, and the meaning of the other variables has already been defined.

### 3.3. Calibration

**Figure 6:**
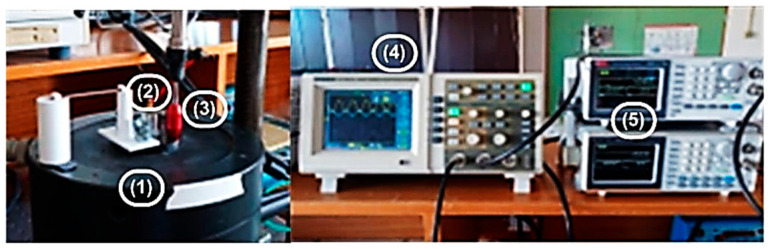
Devices used for calibration purposes: (1)—vibration exciter; (2)—reference accelerometer; (3)—pneumatic cylinder; (4)—digital oscilloscope; (5)—frequency generators.

Regarding measurement system calibration, Figure 6 depicts the calibration setup that was used to perform an initial static and dynamic characterization of the pressure sensors. The main devices used for calibration purposes include an accelerometer [31], a vibration exciter [32], a pneumatic cylinder [33], a digital oscilloscope [34], and two arbitrary waveform generators [35]. The pneumatic cylinder is mechanically coupled with the vibration exciter, whose acceleration, velocity, and positioning are measured by a reference accelerometer, and it generates the pneumatic reference signal used for calibration purposes.

**Figure 7:**
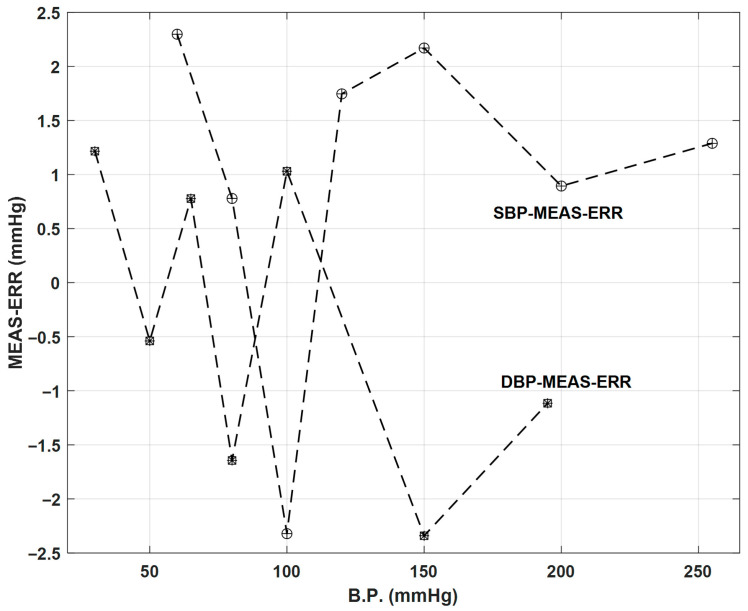
Calibration results of SBP (circle plus symbols) and DBP (circle asterisk symbols) measurements for SBP = {80; 100; 120; 150; 200; 255} mmHg and DBP = {30; 50; 65; 80; 100; 150; 195} mmHg.

The proposed calibration setup enables static and dynamic calibration not only of the pressure sensors but also of the associated pneumatic connecting tubing, which is determinant in terms of measurement system bandwidth limitations. Assuming that an ideal gas behavior approximation is valid [36], the relationship that exists between pneumatic cylinder positioning and the pressure values used for calibration purposes is given by

(3) $$P_{\mathrm{cal}}=\frac{p_{0}}{1-\left(\frac{S_{C}}{S_{\mathrm{PT}}}\cdot \frac{{\Delta l}_{C}}{L_{\mathrm{PT}}}\right)}$$

where P_cal_ represents the pressure value used for calibration purposes, P_0_ represents the initial gas pressure inside the pneumatic tube, SC represents the pneumatic cylinder cross section, SPT represents the pneumatic tube cross section, Δl_C_ represents the pneumatic cylinder displacement relative to the cylinder reference position, and L_PT_ represents the pneumatic tube length. It is important to underline that absolute pressure values must be considered in the previous relationship. Regarding auto-calibration capabilities, the usage of the NIBP and the pressure calibrator enable an automatic calibration of the proposed measurement system. Comparing the measurement results that are obtained with the results obtained by the calibrators and using dummy devices for the cuff and the respiration belt, the EPPR can synthesize a set of reference signals, emulating the real BP and BR signals and making it possible to evaluate the measurement errors. Regarding calibration results, Figure 7 represents the measurement errors associated with SBP and DBP measurements. The absolute errors that are represented correspond to the average errors between the results obtained from the proposed measurement system and results obtained from a previously certified and calibrated BP simulator. The calibration was performed using three complete calibration cycles for the BP values pre-defined in the BP simulator. Regarding BP calibration results, it is verified that the maximum absolute error is lower than 2.5 mmHg, which corresponds to a maximum relative error almost equal to 1.2% for the 225 mmHg BP measuring range.

**Figure 8:**
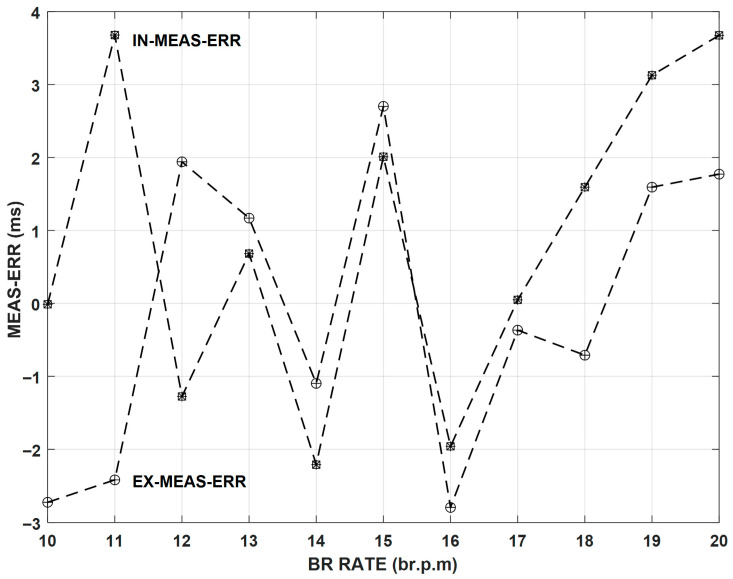
Calibration results of EX duration measurements (circle plus symbols) and IN duration measurements (circle asterisk symbols) for an emulated BR_R varying between 10 and 20 br.p.m, with 1 br.p.m increments (IN/EX = 1).

Regarding BR parameter calibration, Figure 8 represents the measurement errors that were obtained when the EPPR generated a set of triangular waveforms with periods that varied between 3 s and 6 s with 0.3 s increments, which correspond to BR_R varying between 10 and 20 br.p.m with 1 br.p.m. increments, respectively. Regarding BR calibration results, it is verified that, in this case, the maximum absolute error is lower than 4 ms, which corresponds to a maximum relative error always lower than 0.2%.

## 4. Results

This section includes four parts: the first one is related to the presentation of the experimental protocol that was used to evaluate the performance of the measurement system; the second part includes experimental results of stand-alone BP and BR measurements; the third part includes experimental results of correlations that were established between BP and BR parameters; and the four part includes a results discussion. Regarding the correlation method, a bivariate correlation was used, i.e., the correlation between the different variables expressed by a Pearson’s matrix of correlation coefficients [37].

### 4.1. Experimental Protocol

A well-defined experimental protocol was used to improve the strengths of our study by making the experimental tests reliable, reproducible, transparent, and scientifically credible. The same protocol was used for stand-alone BP and BR measurements and for simultaneous measurements of both variables. In this way, it was possible to perform a correct comparative evaluation of the different BP and BR parameters. Two different groups of subjects, which were submitted to a common set of physical exercises, were considered. It is important to note that, for BP measurements, an appropriate cuff size was selected for each subject and that the cuff was wrapped around the bare skin and positioned above the bend of the elbow, at heart level [38]. During measurement, the arm was resting on a flat table surface and any clothing constricting the arm was removed. It is also important to underline that each subject under test remained relaxed and motionless, avoiding any access to their own online measurement data to ensure that measurements were not affected subconsciously by the knowledge of their own measurement data. The experimental tests were performed using two groups of students, each one with ten participants [39]. All participants in this study were previously submitted to a complete medical evaluation to assure their regular health conditions regarding BP and BR parameters. Each group of students included ten males and their age ranged between 18 and 23 years old. One student group regularly practiced physical exercises [40] on a basis of three gymnastic sessions per week, each with a one-hour duration, and the other student group only included subjects that did not practice any kind of regular gymnastic exercises. The test cycle that was considered for each group of students included two steps: in the first step, every subject was submitted to a resting period of 30 min, after which BP and BR measurements were performed; the second step included a 20 min practice of physical exercises, after which a new set of BP and BR measurements were performed. The physical exercises were performed using a motorized treadmill whose speed and inclination were adjustable [41]. This equipment, with wireless communication capabilities, enables a velocity variation between 0.5 km/h and 25.0 km/h, with 0.1 km resolution, and an inclination variation between 0% and 19%, with 1% resolution. The designed physical exercises were executed for 20 min and consisted of a single cycle with the following treadmill settings for exercise practice: a linear velocity variation between 3 and 9 km/h and a treadmill inclination of 10%.

### 4.2. Stand-Alone Measurements

**Figure 9:**
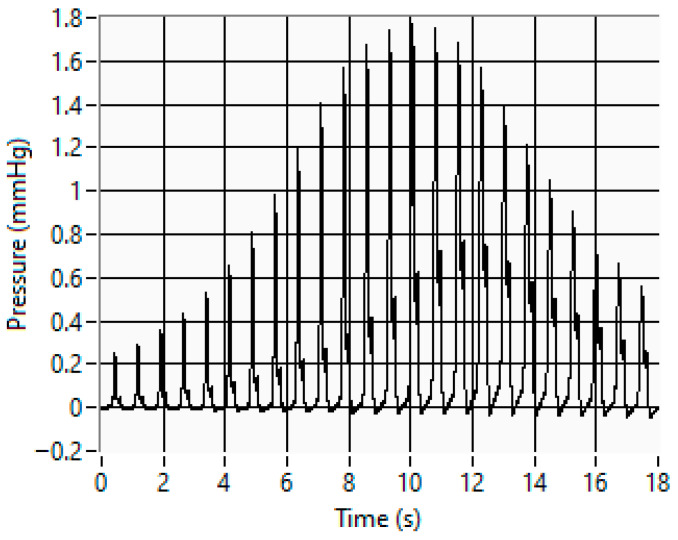
BP oscillometric pulses obtained with the proposed MS for a systolic BP of 127.4 mmHg and a diastolic BP of 83.6 mmHg (MAP = 98.2 mmHg).

This section presents several experimental results of stand-alone measurements of BP and BR parameters. The measurement errors that were calculated were obtained by comparing the experimental results, obtained with the proposed MS, with the measurement results that were obtained with more accurate devices, namely, the BP simulator and the pressure calibrator. As represented in the MS block diagram, the evaluation of the measurement errors associated with BP parameters uses as a reference the measurement results that were obtained with a non-invasive blood pressure simulator (DATREND). The main specifications of this simulator include the capability to generate regulated pressure signals in the range between 0 and 400.0 mmHg, with an accuracy of ±0.5 mmHg and a resolution of 0.1 mmHg. To evaluate pressure measurement errors associated with BR parameters, a reference pressure calibrator (DPI 800) was used. The main specifications of this pressure calibrator include a relative pressure measuring range between 0 and 2 bar (1 bar = 10^5^ Pa), an accuracy better than 0.1% of FS, and 5-digit resolution. This device also enables a continuous measurement of maximum, minimum, and average values of the applied pressure. This capability is particularly advantageous to minimize measurement errors caused by random noise effects. As an example of stand-alone BP measurement results, Figure 9 represents the oscillometric pulses associated with a systolic BP of 127.4 mmHg, a diastolic BP of 83.6 mmHg, and a HBR of 75.4 b.p.m. The measurement configuration associated with the displayed results was a maximum cuff inflation pressure equal to 160 mmHg and minimum cuff deflation pressure equal to 60 mmHg.

**Figure 10:**
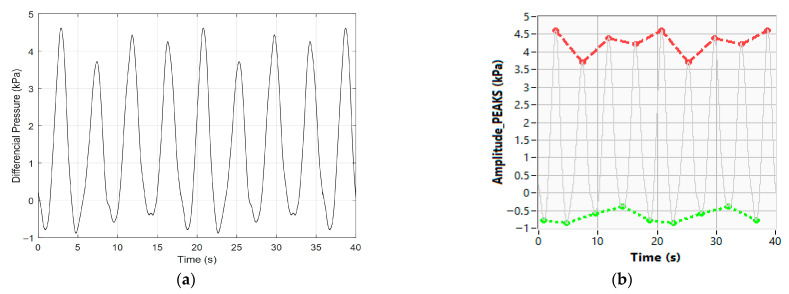
Breathing signal: (**a**) respiratory belt pressure variations caused by thorax volume variations and (**b**) pressure peak amplitudes. red line—maximum peaks envelope; green line—minimum peaks envelope.

In this example, and after a set of ten consecutive measurements, the measurement values obtained by using the reference BP simulator were 125.2 mmHg for the SBP, 85.1 mmHg for the DBP, and 75.2 b.p.m. for the HBR. Thus, the maximum absolute and relative errors for BP measurements, in this case, are equal to 2.2 mmHg and 1.7%, respectively. Regarding the HBR measurement, the maximum absolute error between the proposed MS and the BP simulator, which measures 75.4 b.p.m, gives maximum absolute and relative errors equal to 0.2 b.p.m. and 0.3%, respectively. It is important to note that for calibration purposes, the BP and BR pressure signals were generated by the EPPR device included in the MS block diagram (Figure 3). A more complete calibration with a larger number of BP parameter combinations gave relative error values lower than 2% and 0.5% for BP and HBR measurements, respectively. Regarding stand-alone BR measurements, Figure 10a represents, as an example, the pressure signal acquired through the respiratory belt, and the positive and negative pressure peaks occur at the end of each inhalation and the exhalation, respectively. From Figure 10b it is possible to obtain the duration of the different inhalation and exhalation periods, as well as the maximum and minimum pressure values verified in each breathing cycle. Using Equation (3), it is also possible to obtain, from the pressure peak amplitudes, the maximum air volume compression inside the breathing belt, which in this case is about 3.8% of the air volume contained in the respiration belt without applied mechanical tension. This value can be used as a reference to detect if respiratory belts are too tight or too loose, and this information is very important since both situations cause inaccurate breathing data and alter breathing patterns.

**Figure 11:**
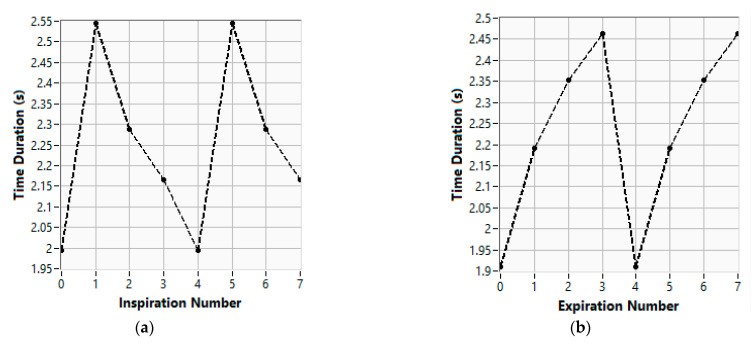
Duration of the inhalation and exhalation periods contained in the breathing signal. Consecutive breathing phase durations: (**a**) inhalation; (**b**) exhalation.

From previous data it is also possible to obtain the BR depth, which corresponds to the peak-to-peak amplitudes of the pressure variations, and from the time duration of the inhalation and exhalation periods, it is also possible to evaluate the BRS, BRD, and BRRg parameters previously defined in Equation (2). Figure 11a and Figure 11b represent the durations of the inspiration and expiration periods, respectively, for the same BR signal that was previously considered.

Using the developed software for breathing signal analysis, it is possible to obtain from the breathing data the maximum, minimum, average, and standard deviation values of the different breathing parameters. Regarding BR calibration, a comparative analysis of the measurement results obtained with the developed prototype and a reference BR analyzer [42] gave, for the different BR parameters, a relative error that is always lower that 4%, which is also acceptable for the MS application purposes.

### 4.3. Correlation Measurements

This subsection includes correlation results between different BP and BR parameters. The experimental protocol used for BP and BR measurements also includes two steps, as referred to in Section 4.1. Regarding label meanings that are associated with the experimental results, measurements obtained after the initial resting period of 30 min are identified with a “before” label, and measurements obtained after the students performed a set of common physical exercises are identified with an “after” label. Regarding correlation coefficients, strong correlations were found between different parameters and for the same parameter before and after the practice of physical exercises. As an example, Table 2 summarizes the correlation results associated with the HBR parameter for the two student groups under analysis (“gym” and “ngym”) before and after the practice of the common set of physical exercises (“before” and “after”). In this example the correlation of the HBR parameter after and before exercise practice is higher than 0.9 for both groups of students, with associated *p*-values lower than 0.01, which means that a statistically significant correlation exists in both cases. It is important to note that the correlation results were evaluated by using a two-tailed significance test and a *p*-value equal to 0.01.

**Figure 12:**
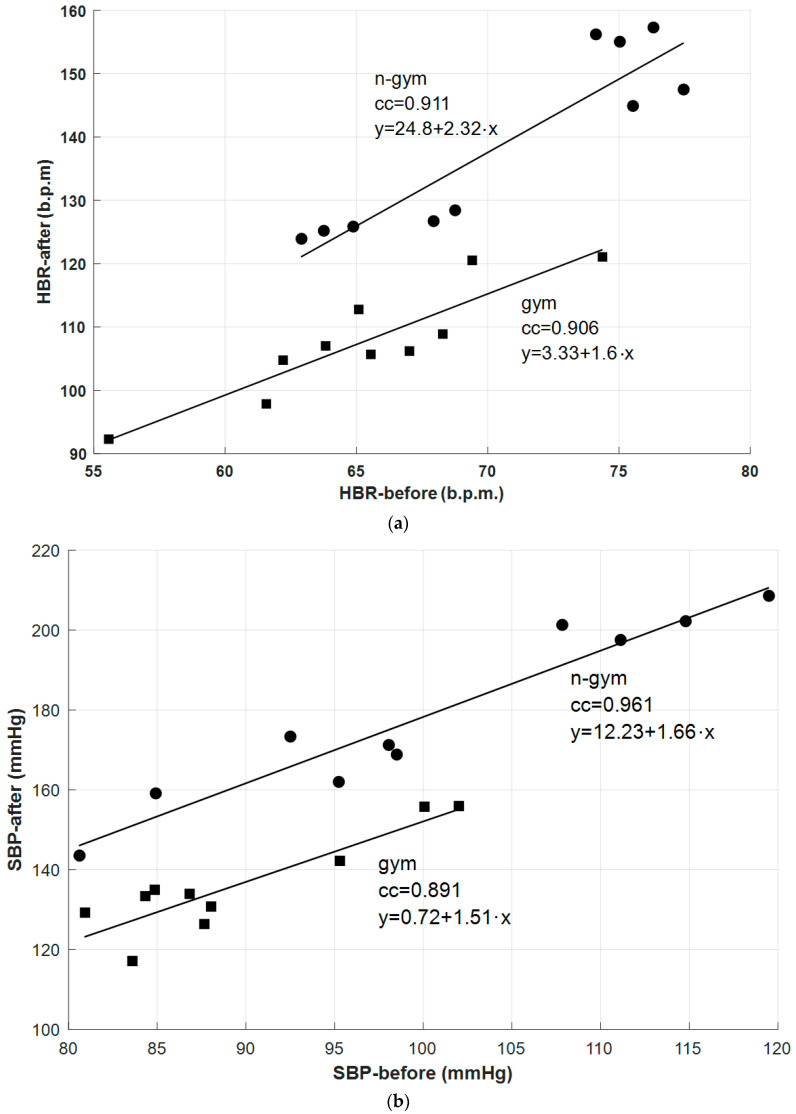
Scatter diagrams and associated linear regression lines before and after the practice of physical exercises for the two groups of students: (**a**) HBR parameter and (**b**) SBP parameter.

Figure 12a and Figure 12b are the graphical representation of the scatter diagrams and the associated linear regression lines for the correlations between the following parameters: HBR-after and HBR-before, and SBP-after and SBP-before, respectively.

**Figure 13:**
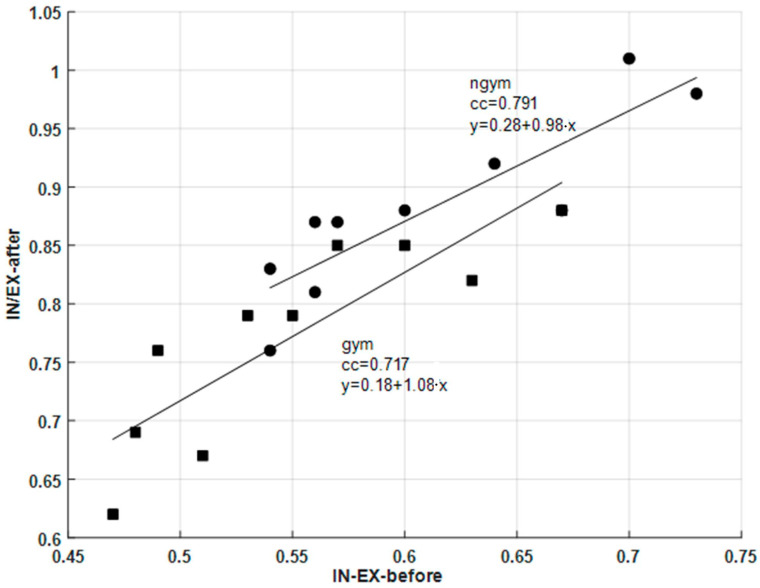
IN/EX ratio parameter scatter diagrams and associated linear regression lines before and after practicing physical exercises for the two groups of students.

As expected, from the previous results, it can be verified that the HBR always increases after practicing physical exercises for both groups of students, since the slopes of the linear regression lines are always universally higher. Regarding the two groups of students, the HBR increase is almost 45% higher in the student group that did not practice regular physical exercises than in the group of students that practiced physical exercises regularly. The same type of conclusion is valid for the analysis of the SBP parameter. For both groups of students, SBP also always increased after practicing physical exercises, since the slopes of the linear regression lines are also universally higher; however, for the SBP parameter, the verified increase is 15% higher in the student group that did not regularly practice gymnastic exercises. It is important to note that other studies [43,44,45,46,47,48] performed by other researchers using different methods also confirmed that the group that practiced exercises had a lower increase in the SBP parameter than the one verified for the student group that did not regularly practice physical exercises. Regarding BR parameter correlation, Figure 13 represents, as an example, the scatter diagrams and the associated linear regression lines that were obtained before and after the practice of physical exercises by the two groups of students. The BR parameter that was analyzed, in this case, was the time ratio between inhalation and exhalation durations (IN/EX) [49]. It can be confirmed that there is also a strong positive correlation between both BR variables, but the values of the correlation coefficients are not as high as the values obtained in the previous correlation analysis. Using previous studies [50,51] for comparative purposes, these results are aligned with expectations, since after exercise practice the inspiration-to-expiration ratio increases. At rest, in this case before performing the physical exercises, the typical IN/EX ratio is around 1:2, with the exhalation duration being almost twice the inhalation duration, and after intense physical exercise, this ratio approaches 1:1. Moreover, it can also be verified that the IN/EX ratio increase is more significant for the “ngym” student group, since the average value of IN/EX for the “gym” student group increases from 0.55 to 0.77, and the average value of the same parameter for the “ngym” student group increases from 0.61 to 0.88.

**Figure 14:**
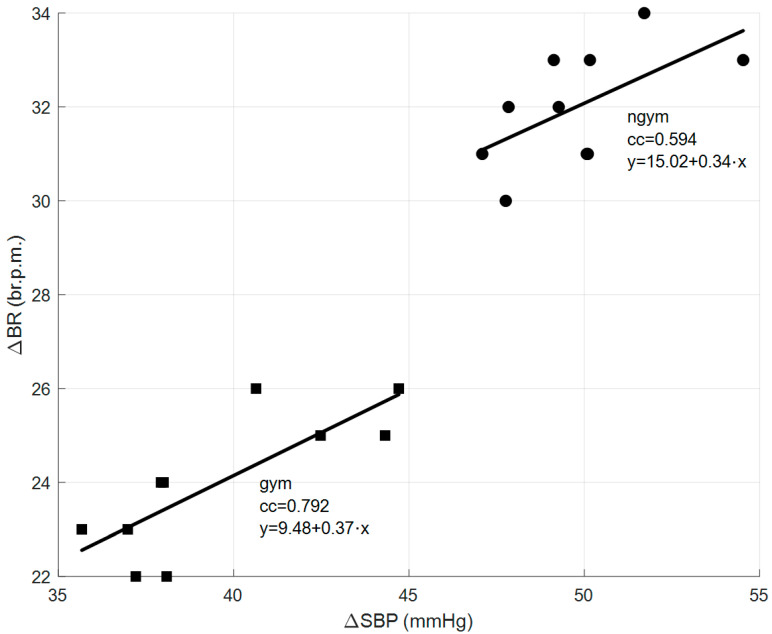
Scatter diagrams and associated linear regression lines for the correlation between the increments of BR and the increments of SBP that are verified after the practicing of physical exercises by the two groups of students.

Finally, regarding cross-correlation between BR and BP parameters, namely, between the increments of breathing rate and the increments of systolic blood pressure, Figure 14 represents, as an example, the scatter diagrams and associated linear regression lines for the correlation between the increments of BR and the increments of SBP that are verified after the practice of physical exercises by the two groups of students.

In this case, it is possible to verify that after practicing physical exercises, for both groups of students, the increment of BR is directly proportional to the increment of SBP, since the slopes of the linear regression lines are always positive. Furthermore, it can be verified that the average values of ΔBR and ΔSBP for the “gym” student group are equal to 24 br.p.m. and 39.6 mmHg, respectively, and the same average values of the increments for the “ngym” student group are equal to 32 br.p.m. and 49.8 mmHg, respectively, which means that the average values of the increments of BR and SBP are always higher for the “ngym” student group, which is as expected due to their lower resistance to physical effort.

### 4.4. Discussion

This subsection quantifies the inter-group differences between the two student groups whose BP and BR parameters were previously analyzed. To determine whether observed differences between experimental groups reflect true effects or are simply due to random variability, *t*-test and F-test inferential tools are used. The *t*-test addresses whether groups differ in central tendency, while the F-test assesses the structure and sources of variability of the experimental data. The combined use of both tests enables us to distinguish true experimental effects from random fluctuations, control false-positive conclusions, and strengthen the scientific validity of the comparisons between the experimental data associated with the gym and ngym groups. Since it is not important to analyze the direction of the effects, a two-tailed *t*-test was considered to compare statistical significance means of the experimental results. Regarding *t*-test result interpretation, the *p*-value that was considered to reject the null hypothesis, i.e., no statistically significant difference between mean values, was a *p*-value lower than 0.05. To interpret the F-test results, the ratio of variances between F and F-critical parameters [37], i.e., the F-ratio in the columns of the table, is considered to evaluate if there is a significant difference between the groups’ means, with this ratio being lower than the one if variances are accepted as similar. Table 3 and Table 4 include a summary of the results that confirm that the observed differences in HBR and SBP increments and IN/EX and BR_SBP increments, respectively, are statistically significant rather than merely numerical differences. As can be easily verified, in the four comparisons that were performed, the *p*-value and F_ratio values are always lower than 0.05 and 1, respectively.

## 5. Conclusions

This paper presented a biomedical measurement system that has dual purposes: the acquisition and processing of BP and BR data. Since the measurements of both variables are performed simultaneously, it is possible to establish real-time correlations between different BP and BR parameters. This feature is interesting for different types of purposes, such as controlling and individualizing the physical exercise intensity for each subject based on their breathing and blood pressure variations before and after practicing physical exercises, hypertension detection and predisposition, pulmonary hypertension detection, and obesity-related respiratory restrictions, among others. A prototype was developed to evaluate the performance of the measurement system and the experimental results that were obtained are consistent with the results obtained by other researchers. The measurement system was calibrated using BP and BR measurement reference devices and the measurement errors that were obtained for different BP and BR parameters were always lower than 1.7% and 2%, respectively. Regarding future work, it is important to improve system performance and to replace the rule-based algorithms used in the joint measurement data processing with predictive algorithms based on ANNs. The generalization of the results, using a larger number of subjects, including people with BP and BR limitations, and the integration of the system with a mobile app to implement longitudinal follow-up studies and to study the long-term value and clinical translation potential of technology must be considered in future developments.

## Figures and Tables

**Table 1:** Comparative analysis of the main advantages and disadvantages of fiber-optic, MEMS, and pneumatic sensing systems for combined BP plus BR parameters measurements.

Technology	Accuracy (BP + BR Measurements)	Cost	Wearing Comfort	Integration Complexity
Fiber-optic	High potential for both if packaging/coupling is well performed; very stable, low drift, EMI/MRI-safe	High (interrogator + specialized components)	Medium (thin/light fiber, but routing + bend sensitivity + robust fixation needed)	High (optical interrogation, packaging, strain/pressure transduction, fiber handling)
MEMS	High for respiration, medium–high for BP depending on method (direct pressure > cuff/patch surrogate); needs calibration and temp compensation	Low–medium (very scalable)	High (small, lightweight; good for wearables)	Low–medium (electronics + packaging + calibration; mature ecosystem)
Pneumatic	High for cuff BP (standard oscillometric method), high for respiration using breathing belts; low–medium for continuous BP waveform (tubing/compliance distortions)	Low–medium (sensors, pumps/valves are generally cheap)	Medium–high (belts comfortable; cuffs can be uncomfortable if frequent inflations are necessary)	Medium–high (leak-proof air path, pumps/valves, tubing dynamics, condensation management)

**Table 2:** Correlation coefficients between the HBR parameter for the two student groups (“gym” and “ngym”) before and after the practice of the physical exercises (“before” and “after”).

		HBR_initial_gym	HBR_after_gym	HBR_initial_ngym
HBR_after_gym	Pearson Correlation	0.906 **		
	Sig. (2-tailed)	<0.001		
	N	10		
HBR_initial_ngym	Pearson Correlation	0.389	0.559	
	Sig. (2-tailed)	0.267	0.093	
	N	10	10	
HBR_after_ngym	Pearson Correlation	0.392	0.597	0.911 **
	Sig. (2-tailed)	0.262	0.069	<0.001
	N	10	10	10

** Correlation is significant at the 0.01 level (2-tailed).

**Table 3:** T-test and F-test results of HBR and SBP increments for the two student groups (gym and ngym).

ID	GYM	HBR_init	HBR_after	INC_HBR	Mean	Var	*p*	F_ratio	SBP_init	SBP_after	INC_SBP	Mean	Var	*p*	F_ratio
1	1	74	121	47	42.4	23.5	**4 × 10** ^**−7**^	**0.84**	85	135	50	46.6	45.1	**2 × 10** ^**−7**^	**0.32**
2	1	69	121	51					84	117	34				
3	1	62	105	43					100	156	56				
4	1	67	106	39					81	129	48				
5	1	68	109	41					102	156	54				
6	1	62	98	36					88	131	43				
7	1	64	107	43					88	126	39				
8	1	66	106	40					84	133	49				
9	1	56	92	37					87	134	47				
10	1	65	113	48					95	142	47				
11	0	77	147	70	68.4	90.1			119	209	89	78.5	108.6		
12	0	75	155	80					108	201	93				
13	0	76	145	69					81	144	63				
14	0	69	128	60					98	171	73				
15	0	64	125	61					95	162	67				
16	0	65	126	61					99	169	70				
17	0	63	124	61					93	173	81				
18	0	74	156	82					115	202	87				
19	0	68	127	59					85	159	74				
20	0	76	157	81					111	198	86				

**Table 4:** T-test and F-test results of IN/EX ratio and BR_SBP increments for the two student groups (gym and ngym).

ID	IN/EX_init	IN/EX_after	INC_IN/EX	Mean	Var	*p*	F_ratio	D_BR	D_SBP	INC_BR_SBP	Mean	Var	*p*	F_ratio
1	0.51	0.67	0.16	**0.22**	**2 × 10** ^**−3**^	**2 × 10** ^**−2**^	**0.52**	24	38	14	**15.6**	**5 × 10** ^**0**^	**3 × 10** ^**−2**^	**0.50**
2	0.47	0.62	0.15					23	36	13				
3	0.63	0.82	0.19					25	42	17				
4	0.49	0.76	0.27					24	38	14				
5	0.67	0.88	0.21					26	45	19				
6	0.55	0.79	0.24					25	44	19				
7	0.60	0.85	0.25					22	37	15				
8	0.48	0.69	0.21					23	37	14				
9	0.57	0.85	0.28					22	38	16				
10	0.53	0.79	0.26					26	41	15				
11	0.56	0.87	0.31	**0.27**	**1 × 10** ^**−3**^			32	48	16	**17.8**	**3 × 10** ^**0**^		
12	0.56	0.81	0.25					31	50	19				
13	0.73	0.98	0.25					33	50	17				
14	0.60	0.88	0.28					34	52	18				
15	0.70	1.01	0.31					30	48	18				
16	0.57	0.87	0.30					33	55	22				
17	0.67	0.88	0.21					31	47	16				
18	0.54	0.76	0.22					32	49	17				
19	0.64	0.92	0.28					33	49	16				
20	0.54	0.83	0.29					31	50	19				

## Data Availability

The original contributions presented in this study are included in the article. Further inquiries can be directed to the corresponding author.
